# Socioeconomic Inequalities in Oral Health among Adults in Guangxi, China

**DOI:** 10.3290/j.ohpd.b4836051

**Published:** 2024-01-15

**Authors:** Andi Li, Tingting Zhang, Qiulin Liu, Xueting Yu, Xiaojuan Zeng

**Affiliations:** a MS Student, Department of Dental Public Health, College and Hospital of Stomatology, Guangxi Medical University, Nanning, China. Conceived the study and developed the analysis strategy, carried out the analysis, drafted the manuscript, approved the final manuscript.; b MS Student, Department of Dental Public Health, College and Hospital of Stomatology, Guangxi Medical University, Nanning, China. Conceived the study and developed the analysis strategy, drafted the manuscript, approved the final manuscript.; c Lecturer, Department of Dental Public Health, College and Hospital of Stomatology, Guangxi Medical University, Nanning, China. Critically reviewed the drafts and gave text suggestions, approved the final manuscript.; d Lecturer, Department of Dental Public Health, College and Hospital of Stomatology, Guangxi Medical University, Nanning, China. Critically reviewed the drafts and gave text suggestions, approved the final manuscript.; e Professor, Department of Dental Public Health, College and Hospital of Stomatology, Guangxi Medical University, Nanning, China. Conceived the study and developed the analysis strategy, critically reviewed the drafts and gave text suggestions, approved the final manuscript.

**Keywords:** oral health, socioeconomic inequalities, socioeconomic status

## Abstract

**Purpose:**

To examine the relationship between socioeconomic inequalities and oral health among adults in the Guangxi province of China.

**Materials and Methods:**

The present work was designed as a cross-sectional study, and comprises a secondary analysis of the Fourth National Oral Health Survey from 2015–2016. A multistage cluster sampling method was adopted for this survey, conducted in three urban and three rural districts Guangxi province. Dental examinations were conducted to determine oral health indicators: decayed teeth (DT), clinical attachment loss (CAL) and missing teeth (MT). The outcome measures were DT, CAL and MT. A structured questionnaire was used to collect data on demographic characteristics and socioeconomic status (SES). Multiple logistic regression models were used to analyse the relationship between SES and oral health by adjusting covariates.

**Results:**

The sample consisted of 651 participants aged 35–74 years. Logisitic analysis showed a statistically significant association between SES and oral health indicators. In the fully adjusted model, participants with primary education were more likely to suffer more DT (OR = 2.67, 95% CI: 1.17–6.10), teeth with CAL ≥ 4 mm (OR = 2.15, 95% CI: 1.25–3.67) and MT (OR = 3.04, 95% CI: 1.65–5.60) compared to the higher education group. Participants with secondary education exhibited a higher likelihood of experiencing increased MT compared to those in the higher education group in the fully adjusted model (OR = 3.21, 95% CI: 1.78–5.76). Household income was associated with DT and MT in the unadjusted model only.

**Conclusions:**

There was strong relationship between SES and oral health of adults. The survey suggested a relationship between low educational attainment and oral health.

Oral diseases pose a significant public health concern on a global scale, impacting a population of over 3.5 billion individuals.^2^ Globally, poor oral health among adults is reflected particularly in high levels of tooth loss, caries experience, periodontal disease, and oral cancer.^6^^,^^12^ Extensive research has established a strong correlation between oral health and socioeconomic status (SES), highlighting that individuals from disadvantaged backgrounds are at a heightened risk of experiencing oral diseases.^10^^,^^14^ Specifically, those with lower SES are more likely to exhibit poorer oral health outcomes in comparison to individuals with higher SES. For instance, individuals in Australia who have only completed a primary-school education are more than twice as likely to have untreated caries compared to those who have obtained a college degree. This disparity may be attributed to limited access to employment opportunities, lower receptiveness to health messages, and challenges in navigating healthcare systems.^19^

SES parameters such as low educational level and income have been identified as risk factors for oral health and healthcare utilisation.^20^^,^^29^ In the age group of 65-74 years, individuals with a low income were found to have nearly twice the likelihood of caries experience compared to those with a high income. Furthermore, illiterate individuals were more prone to having a higher decayed, missing and filled teeth (DMFT) score in comparison to those with higher educational attainment.^32^ The ability to afford health services and make clinical decisions with long-term implications for oral health can be influenced by income and educational levels, which in turn can impact the choice of health services and information of health.^3^ Two studies have identified socioeconomic inequalities in among children aged 3 to 5 and 12 years in Guangxi.^13^^,^^26^ However, there is a dearth of research focusing on socioeconomic inequalities among adults in Guangxi. Therefore, it seems imperative to investigate this situation further in Guangxi. This study aimed to assess socioeconomic inequalities in oral health in the Guangxi province of China, utilising data obtained from The Fourth Chinese National Oral Health Survey. The study provided updated information employing various SES indicators and oral health measures; this is relevant for supporting public policy recommendations.

## MATERIALS AND METHODS

### Design and Setting

With a cross-sectional design, the current study used data from the Fourth Chinese National Oral Health Survey, 2015–2016. The survey was conducted by the Chinese Stomatological Association in cooperation with the Chinese Centre for Disease Control (CDC). Although the survey methods have been described in detail elsewhere,^18^ here follows as brief summary of the study methodology.

### Sampling

A multistage cluster sampling method was adopted for this survey. Three urban and three rural districts were selected in Guangxi province using probability proportional to size (PPS) sampling. Three subdistricts were then selected using the PPS sampling method in each district. Finally, participants aged 35–44, 55–64, and 65–74 were selected using the quota sampling method.

The following formula was used to calculate the sample size:

$$\text{n}=deff=\frac{\mu_{\text{α}}^{2}p\left(1-p\right)}{{\text{δ}}^{2}}$$

where the design effect deff = 2, the level of confidence µ = 1.96, and the acceptable error δ = 15%*p*. According to the Third National Oral Health Survey in Guangxi province, the prevalence of periodontitis in adults was 90.8%. Considering 8% of the non-response rate, a total of 651 participants were recruited in the study.

### Ethical Approval

The 4th National Oral Health Survey was approved by the Ethics Committee of the Chinese Stomatological Association (Approval no. 2014-003). Written informed consent was obtained from the participants.

### Dental Examination

Dental examinations were conducted by trained and calibrated dentist-examiners based on the criteria of World Health Organization(WHO) Oral Health Survey Basic Methods (5th edition).^33^ Participants were required to sit on a chair for examination, and dental mirrors and a Community Periodontal Index (CPI) probe were used under artificial light. Oral health status, including decayed teeth (DT), clinical attachment loss (CAL) and missing teeth (MT), was collected. Structured questionnaires were used to collect data on demographic characteristics and socioeconomic status.

### Measures

#### Independent variables

Educational level and household income were used as indicators for SES. Educational level was categorised into three groups: (1) primary (less than 6 years); (2) secondary (7 to 9 years); (3) higher (10 years or more). Number of family members was also collected, as well as household income. Average household income was divided into three groups: low (less than ¥3000/year), medium (¥3000/year to ¥8000 /year), and high (more than 8000 ¥/year).

#### Dependent variables

Three oral health outcomes were used. (1) The number of DT was categorised according to two levels of severity: high caries (DT ≥ 7) and low caries (DT < 7); (2) the number of teeth with CAL ≥ 4 mm was classified into two levels: poor periodontal status (at least one tooth with CAL ≥ 4 mm) and fair periodontal status (CAL < 4 mm); (3) the number of MT was categorised as two groups: more missing teeth (MT ≥ 5) and few missing teeth (MT < 5).

#### Covariates

Age, gender (male/female), ethnicity (Han/other ethnicities), place of residence (urban/rural), sugar intake daily (more than once/less than once), daily toothbrushing (at least twice/once or less), dental attendance (yes/no), smoker (yes/no), and self-perceived general health (good/fair/poor) were considered as covariates to exclude the potential effect of these factors.

### Statistical Analysis

Data were analysed using SPSS 22.0 (IBM; Armonk, NY, USA). Descriptive results were presented as means. The Mann-Whitney U-test (two groups) and the Kruskal-Wallis test were used to assess the relationships between oral health status, SES and covariates. Bivariate and multiple logistic regressions were performed to determine whether the SES indicators were associated with oral health status. Multiple logistic regressions were conducted in a model adjusted for demographics, oral health-related behaviours, self-perceived general health. There were three models in the multiple logistic regression analysis. In these models, variables were added stepwise to explore the effects of SES on oral health status after adjusting covariates. Model 1 was a crude model for each of the variables. Demographics were adjusted in Model 2 to confirm whether these covariates influence the association between oral health outcomes and SES. Finally, oral health-related behaviour and self-perceived general health were added to Model 3. The odds ratios (ORs) and 95% confidence intervals (95% CIs) were calculated. In all the analyses, the level of statistical significance was set at p < 0.05.

## RESULTS

A total of 651 adults aged 35–74 years were recruited for this study. The prevalence of high caries (DT ≥ 7), poor periodontal status (at least one tooth with CAL ≥ 4 mm) and more missing teeth (MT ≥ 5) was 70.0%, 48.7% and 85.7%, respectively. The personal mean number (± SD) of DT, teeth with CAL ≥ 4 mm, and MT were 2.86 ± 3.74, 2.25 ± 3.80, and 4.66 ± 5.51, respectively (Table 1).

**Table 1:** Basic characteristics of oral health outcomes in the study participants

Oral health outcome	N (%)	Mean ± SD
DT ≥ 7	456 (70.0)	2.86 ± 3.74
CAL ≥ 4 mm	317 (48.7)	2.25 ± 3.80
MT ≥ 5	558 (85.7)	4.66 ± 5.51

Demographics, socioeconomic status, oral health-related behaviours and self-perceived general health are summarised in Table 2. In addition, there was a statistically significant association between oral health and socioeconomic status, with a few exceptions regarding household income (Table 2).

**Table 2:** Summary of the characteristics of the study participants

Variables	n	%	DT (Mean ± SD)	P	CAL ≥ 4 mm (Mean ± SD)	p	MT (Mean ± SD)	p
**Age (years)**								
35–44	216	33.2	1.55 ± 2.60	0.001*	0.47 ± 1.38	0.001*	2.06 ± 1.72	0.001*
55–64	218	33.5	3.04 ± 3.53		2.97 ± 4.06		4.43 ± 4.78	
65–74	217	33.3	3.98 ± 4.45		3.30 ± 4.49		7.47 ± 7.12	
**Gender**								
Male	323	49.6	2.60 ± 3.47	0.018*	3.18 ± 4.56	0.001*	4.44 ± 5.02	0.729
Female	328	50.4	3.11 ± 3.98		1.34 ± 2.55		4.88 ± 5.95	
**Ethnicity**								
Han	419	64.4	2.83 ± 3.76	0.453	2.56 ± 3.93	0.006*	4.52 ± 5.23	0.875
Other ethnicities	232	35.6	2.91 ± 3.70		1.71 ± 3.49		4.91 ± 5.98	
**Place of residence**								
Urban	326	50.1	2.52 ± 3.63	0.002*	2.67 ± 4.54	0.547	4.37 ± 5.19	0.282
Rural	325	49.9	3.20 ± 3.82		1.83 ± 2.80		4.95 ± 5.81	
**Educational level**								
Primary	256	39.3	3.68 ± 4.16	0.001*	2.37 ± 3.42	0.001*	6.11 ± 6.89	0.001*
Secondary	212	32.6	2.76 ± 3.77		2.63 ± 4.50		4.41 ± 4.68	
Higher	183	28.1	1.81 ± 2.67		1.66 ± 3.32		2.92 ± 3.21	
**Household income** (CNY)								
Low	217	33.3	3.21 ± 3.88	0.022*	1.84 ± 2.82	0.510	4.78 ± 5.65	0.793
Middle	210	32.3	3.10 ± 4.13		1.95 ± 3.12		4.99 ± 6.02	
High	224	34.4	2.29 ± 3.11		2.94 ± 4.95		4.23 ± 4.82	
**Sugar intake daily**								
More than once	75	11.5	2.56 ± 2.90	0.947	3.01 ± 5.66	0.805	4.73 ± 5.07	0.352
Less than once	576	88.5	2.90 ± 3.84		2.15 ± 3.48		4.65 ± 5.57	
**Toothbrushing daily**								
At least twice	303	46.5	2.27 ± 3.36	0.001*	2.22 ± 4.09	0.028*	4.12 ± 5.36	0.001*
Once or less	348	53.5	3.37 ± 3.97		2.28 ± 3.53		5.14 ± 5.60	
**Dental attendance**								
Yes	364	55.9	2.99 ± 3.68	0.046*	1.97 ± 3.34	0.172	5.51 ± 6.29	0.001*
No	287	44.1	2.70 ± 3.82		2.61 ± 4.28		3.58 ± 4.07	
**Smoker**								
Yes	143	22.0	2.44 ± 3.31	0.086	3.17 ± 4.43	0.001*	5.15 ± 5.74	0.076
No	508	78.0	2.98 ± 3.85		2.00 ± 3.56		4.52 ± 5.44	
**Self-perceived general health**								
Good	169	26.0	2.16 ± 2.99	0.001*	2.01 ± 3.37	0.613	3.65 ± 4.07	0.007*
Fair	409	62.8	2.77 ± 3.36		2.30 ± 3.96		4.75 ± 5.64	
Poor	73	11.2	5.00 ± 5.97		2.53 ± 3.81		6.49 ± 7.00	

**Fig 1:**
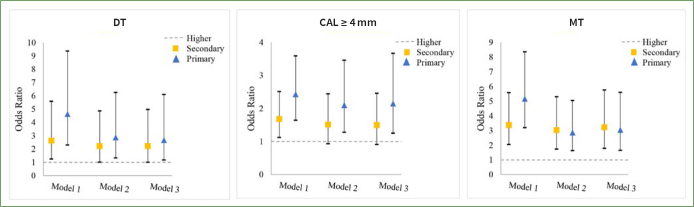
Odds ratios (ORs) of DT, CAL ≥ 4 mm and MT by educational level. p < 0.05 (trend). Model 1: crude model. Model 2: adjusted for educational level, age, gender, ethnicity, place of residence. Model 3: adjusted for educational level, age, gender, ethnicity, place of residence, daily sugar intake, daily toothbrushing, dental attendance, smoker and self-perceived general health.

In the logistic regression model, a statistically significant relationship was observed between DT and education level. Figure 1 illustrates that, in Model 1, participants with primary (OR = 4.63, 95% CI: 2.29–9.36) or secondary (OR = 2.63, 95% CI: 1.24–5.58) education exhibited statistically significantly more dental caries compared to the higher education group. After adjusting for household income, age, gender, ethnicity, and place of residence (Model 2), the ORs for the primary (OR = 2.87, 95% CI: 1.32–6.25) and secondary (OR = 2.22, 95% CI: 1.01–4.86) education remained statistically significant. In the fully adjusted model (Model 3), the OR for participants with primary education remained statistically significant (OR = 2.67, 95% CI: 1.17–6.10), whereas OR for secondary education did not differ from higher education (OR = 2.22, 95% CI: 1.00–4.97) (Fig 1).

In relation to CAL ≥ 4 mm, participants with primary education showed a statistically significantly higher OR compared to those in the higher education group in Model 1 (OR = 2.43, 95% CI: 1.64-3.59), Model 2 (OR = 2.10, 95% CI: 1.28-3.46), and Model 3 (OR = 2.15, 95% CI: 1.25–3.67). Conversely, participants with secondary education displayed statistically significantly higher ORs compared to those with higher education in Model 1 (OR = 1.68, 95% CI: 1.12–2.51). However, after adjusting for covariates in Models 2 and 3, statistically significant ORs were no longer found in secondary education (Fig 1).

Regarding MT, logistic regression analysis revealed that participants with primary or secondary education exhibited a higher likelihood of experiencing increased MT, even after adjusting covariates (Models 1 to 3).

**Fig 2:**
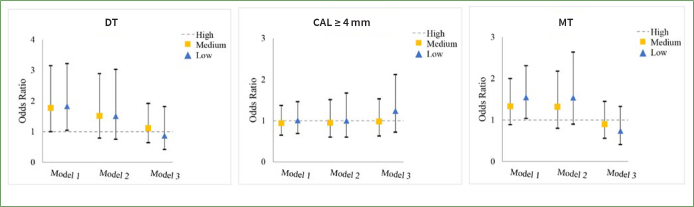
Odds ratios (ORs) of DT, CAL ≥ 4 mm and MT by household income. p < 0.05 (trend). Model 1: crude model. Model 2: adjusted for educational level, age, gender, ethnicity, place of residence. Model 3: adjusted for educational level, age, gender, ethnicity, place of residence, daily sugar intake, daily toothbrushing, dental attendance, smoker and self-perceived general health.

As to the relationship between oral health outcome and household income, it was observed that the ORs for DT (OR = 1.83, 95% CI: 1.04–3.22) and MT (OR = 1.55, 95% CI: 1.04–2.31) were notably higher in low-income households compared to high-income households in Model 1. However, after adjusting for covariates, statistical significance was not observed in Models 2 and 3 (Fig 2).

These data may be accessed on upon contacting the corresponding author. The same principle applies for the statistical analysis script.

## DISCUSSION

Our study provides empirical evidence that socioeconomic inequalities in oral health exist among adults in Guangxi, China. Specifically, individuals with lower education were more likely to experience untreated caries, clinical attachment loss and missing teeth. Univariate analysis revealed a statistically significant association between low SES and oral health. However, logistic regression analysis did not demonstrate a statistically significant increased risk with lower household income. This discrepancy suggests potential inaccuracies in self-reported household income in the questionnaire. Nevertheless, our findings reinforce the plausible interpretation of the effect of disadvantaged SES on oral health. Additionally, we found that educational attainment was significantly associated with oral health. Despite adjustments for oral health-related behaviours and self-perceived general health, these associations were weakened but not eradicated.

Consistent with the existing literature, our findings demonstrate that individuals with a higher education tend to exhibit superior oral health. Furthermore, the link between low SES and poor oral health has been confirmed in both cross-sectional and longitudinal investigations.^16^^,^^17^^,^^21^^,^^30^ The effect of SES on oral health encompasses factors such as dental health utilisation, health diffusion and psychological stress.^25^ In addition, individuals who pursue education can experience greater health benefits.^34^ For instance, individuals with higher education tend to possess greater personal health knowledge and better health attitudes, which in turn leads to increased initiative in terms of dental visits, access to information on preventively maintaining/improving oral health, and thus improved oral health. In addition to income, education was also the crucial contributor to oral health inequalities.^9^ A cross-sectional study conducted in Lebanon confirmed that the presence of socioeconomic inequalities existed in Lebanese older people, which could be attributed to behavioural factors.^8^^,^^9^ Nonetheless, a study on Brazilian adults did not show that educational level is an important factor in oral health.^27^

Another salient finding is the inequality of periodontal status. Our study found that individuals with low SES may be at a higher risk of poor periodontal status in adulthood. This observation aligns with the current understanding of periodontitis and its underlying aetiology. A systematic review showed that individuals with low SES are more likely to develop and experience progression of periodontitis.^23^ Additionally, a meta-analysis of nine cross-sectional studies and two cohort studies revealed a statistically significant association between low income and tooth loss.^25^ Among adults in Valencia, Spain, the odds ratio for low SES in the presence of periodontal pockets was 1.81, indicating a statistically significant association between low SES and a higher prevalence of periodontal disease in the adult population.^1^ However, a 10-year-cohort study did not report a statistically significant relationship between SES and periodontitis.^22^

It was interesting that we did not find a statistically significant relationship between household income and oral health, which was in line with some current studies.^4^^,^^7^^,^^15^ A cross-sectional national study conducted in Brazil suggested that income inequality was not associated with the occurrence of cumulative dental caries (edentulism) or any periodontal diseases.^4^ Meanwhile, there was no discernible relationship between the prevalence of functional dentition and household income.^5^ Moreover, a pooled median DMFT score among 35- to 44-year-olds was 13.5 in the high-income countries, whereas in the low-income countries, it was 3.111, which contradicts the prevailing literature on this topic. However, numerous studies have assessed relationships between low household income and oral health.^7^^,^^15^ A pooled odd ratios of 1.40 (95% CI: 1.19 to 1.65) was identified from 15 studies, indicating a statistically significant association between low income and caries experience.^24^ Tooth loss is also strongly linked to low income. Material factors and behavioural/cultural factors are commonly-given reasons for the relationship between low income and worse oral health.^28^^,^^31^ Material disadvantage plays an important role, as individuals with limited financial resources may face challenges in accessing preventive and regular dental services due to the high costs associated with treatment and the unavailability of nutritious diets. Furthermore, behavioural/cultural parameters emphasise the importance of poor health behaviours such as smoking, high sugar consumption, irregular dental visits and inadequate oral hygiene routines, which may be caused by low income.

This study is not free of limitations. Firstly, due to privacy concerns surrounding household income, our analysis was restricted in its ability to accurately capture household income, which lead to the statistically non-significant result of income inequality. Secondly, the cross-sectional analysis cannot examine how socioeconomic inequalities in oral health changed over time, which reduces the evidence of potential causal pathways in oral health inequalities. Finally, occupation could provide a more comprehensive assessment of an individual’s socioeconomic status, encompassing both educational attainment and household income.

Nevertheless, our study possesses strengths that warrant consideration. To the best of our knowledge, this is the first cross-sectional study revealing the association between SES and oral health in adults residing in Guangxi, China. Moreover, the rigorous scientific sampling progress provided strong and representative evidence. These aspects compensate the small number of participants included in this study. Furthermore, the inclusion of studies from different settings suggests that the association between educational attainment and adult oral health is a global concern. Consequently, our study contributes to the existing body of knowledge in this field.

Our study has important policy implications. On the one hand, the positive effect of increased individual educational attainment on adult oral health implies that government subsidy programmes targeting those with little education in oral health may be an effective way to improve oral health of adults with low educational levels. On the other hand, given the importance of income in oral health, this implies a potential need to reduce income inequality.

## CONCLUSIONS

This study confirms a strong association between SES and adult oral health, proving that socioeconomic inequalities affect adult oral health in Guangxi, China. Despite the limitations of this study, the observed statistically significant association between SES and adult oral health cannot be dismissed. It is recommended that future research employ longitudinal designs and incorporate measures of income and occupation to further validate our findings.
